# Boerhaave syndrome: Successful conservative treatment. Case report and literature review

**DOI:** 10.1016/j.ijscr.2023.108289

**Published:** 2023-05-04

**Authors:** Maria Alejandra Díaz Tarazona, Carlos Eduardo Rey Chaves, Juan Felipe Infante Mateus, Francisco Alejandro Rincón Comba, J.D. Rosso, Maria Camila Azula Uribe

**Affiliations:** a Estudiante de posgrado Cirugía General, Pontificia Universidad Javeriana, Facultad de Medicina, Bogotá, Colombia; b Cirujano General, Cirugía General, Pontificia Universidad Javeriana, Facultad de Medicina, Hospital Universitario San Ignacio, Bogotá, Colombia; c Médico General, Hospital Universitario San Ignacio, Bogotá, Colombia

**Keywords:** Esophageal perforation, Esophageal diseases, Boerhaave syndrome, Conservative

## Abstract

**Introduction and importance:**

Spontaneous esophageal perforation or “Boerhaave” syndrome is an uncommon pathology, with high rates of morbidity and mortality. Clinical scores such as the Pittsburgh classification could guide the treatment and helps to assess mortality risk. Conservative management could be performed in selected cases.

**Case presentation:**

We present a 19-year-old male patient with a previous history of anxiety and depression, who enters the emergency room with vomiting and epigastric pain followed by swelling at the neck and dysphagia. Neck tomography and chest tomography were obtained showing subcutaneous emphysema. Conservative management was indicated and after 10 days of in-hospital stay and no complications, the patient was discharged. Any complication was observed after 30, 60, and 90 days of follow-up.

**Clinical discussion:**

Selected patients with Boerhaave syndrome could benefit from conservative management. Risk classification could be performed using the Pittsburgh score. Nil per os, antibiotic treatment, and nutritional support are the cornerstone of nonoperative management.

**Conclusion:**

Boerhaave syndrome it's an infrequent pathology, with mortality rates ranging between 30 and 50 %. Early identification and on-time management are required to have favorable outcomes. Pittsburgh score can be used to guide the selection of patients who benefit from conservative treatment.

## Highlights

• Boerhaave syndrome remains to be an uncommon pathology with high rates of morbidity and mortality.
• Early recognition leads to appropriate management and favorable results.
• Pittsburgh score helps to adequate classification of the patients according to mortality risk.

## 1 Introduction

Boerhaave syndrome (BS) or spontaneous esophageal perforation was first described by Herman Boerhaave in 1729 [1]. It's an uncommon but potentially lethal condition related to an esophageal tear related to an increased esophageal pressure in absence of trauma or iatrogenic injury [1], [2]. Mortality rounds 30 to 50 % in some case series [1], [2], [3], and the vast majority of the patients require surgical management with high morbidity rates, nevertheless, nonoperative management could be performed in selected patients [1], [3], [4].

Traditionally patients presented to the emergency room with chest pain, vomiting, and subcutaneous emphysema and constituted the named Macler's triad, nevertheless, this triad it's only evidenced in 14 % of the population [1], [2], [3], [4], [5]. Thus, reflects that BS is a diagnostic challenge due to non-specific signs and symptoms [6]. Diagnostic imaging modalities that could be performed include contrast tomography (CT) with acceptable sensitivity and specificity (92–100 %) or an esophagogram that could show important information regarding the location and characteristics of a possible leak [1].

Favorable outcomes depend on early recognition of the condition and avoiding delay in operative management when required [1], [6]. However, there is a lack of literature regarding this condition, and there is no standardized specific management for patients with BS [7].

The aim of this article is to present a case of spontaneous esophageal perforation in Colombia, in which non-operative management was successful.

## 2 Presentation of the case

After ethical and institutional approval, previous informed consent was filled, following SCARE guidelines [8]. We present a 19-year-old woman, who presents to the emergency room with 3 months of intermittent postprandial vomiting episodes (12 episodes daily); in the last 24 h refers to high-intensity abdominal pain located in an epigastric irradiated to the retrosternal region. Posteriorly with swelling in the anterior area of the neck and dysphagia. As a personal history, of depression and anxiety, in treatment with quetiapine and fluoxetine. There is no previous relevant history of gastrointestinal disease.

Initial assessment by general surgery was performed, with normal vital signs (heart rate: 84 beats/min, respiratory rate 16/min, blood pressure: 100/64 mm Hg), with extensive subcutaneous emphysema located at the submandibular region, anterior area of the neck to the second intercostal space. With mild pain at epigastric palpation.

**Fig. 1:**
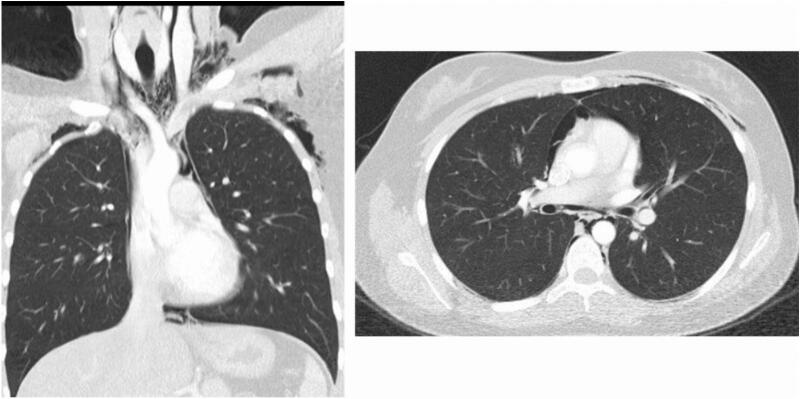
Chest CT scan. Pneumomediastinum.

**Fig. 2:**
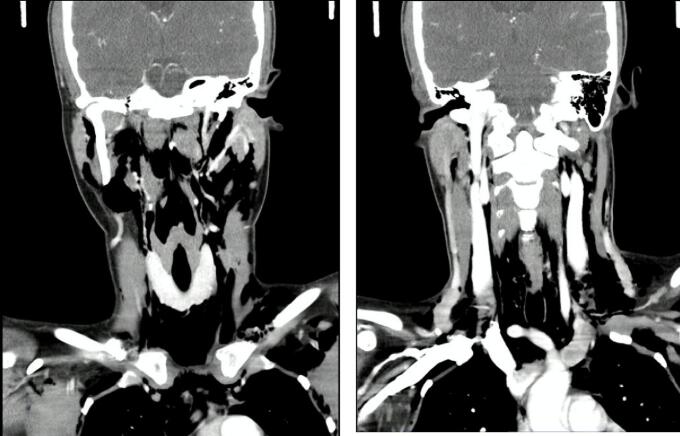
Neck CT scan. Subcutaneous emphysema through the dangerous space.

It was decided to request a chest CT (Fig. 1) that shows extensive pneumomediastinum and a neck CT (Fig. 2) that evidenced emphysema that compromises the thorax, neck, and face, however without signs of mediastinitis. A complete blood count was performed, mild leukocytosis, neutrophilia, no anemia, or thrombocytopenia (white blood cell count 11,500 neutrophils: 91.8 % hemoglobin: 14.4 g/dl platelets: 423,100).

Pittsburgh's score was calculated with 1 point, and for that reason, medical management was indicated. Nil per os, and parenteral nutritional support was initiated, analgesia, proton pump inhibitor, and wide spectrum antibiotic were initiated. Psychiatrist consultations were required for the management of anxiety and depression. After 7 days of management, a digestive tract x-ray was performed without evidence of leak; subcutaneous emphysema resolved and tolerance to oral intake was evaluated progressively, with adequate tolerance; after 10 days of in-hospital stay was discharged.

After 30, 60 and 90 days, no complications were evidenced, with normal endoscopic findings.

## 3 Discussion

BS is an uncommon entity with unknown incidence due to sub-diagnosis and in most of the cases, the diagnosis was performed post-mortem. BS shows high rates of mortality that range between 30 and 50 % in some case series and could reach 100 % in the absence of adequate treatment [1], [7]. Perforation is related to a sudden intra-esophageal pressure usually after vomiting [1], [2], [3], and represents <20 % of the causes of esophageal perforation; the most common etiologies include foreign bodies, iatrogenic, trauma-related, inflammatory, or neoplastic pathologies [1], [2], [3], [4], [5], [6], [7].

The location of the perforation it's usually in the lower third of the esophagus (left side most frequently) at 2–4 cm above the gastroesophageal junction due to thinness in the muscle fibers and no local protection with a weak in the esophageal wall [1], [3], [5].

Due to the non-specific clinical signs, and the broad spectrum of clinical presentation, diagnosis is challenging for the surgeon [1], [2], [4]. There is a male predominance in a 2:1 proportion, with a peak of incidence between 50 and 70 years [1], [2], and previous history of alcohol abuse, or psychiatric disorders should be evaluated [1], [6]. In almost 15 % of the patients with BS, Mackler's triad it's evidenced and includes chest pain, vomiting, and subcutaneous emphysema [1], nevertheless, other symptoms include uncontrollable vomiting, abdominal pain, dysphagia, dyspnea, or clinical signs of sepsis related to the progression of the pathology [1]. In our case, clinical triad was evidenced, and diagnosis was suspected due to presence of vomiting, chest pain and subcutaneous emphysema that located in neck and chest.

There is an inherent risk of mediastinitis due to the connection between neck spaces and mediastinum, and for that reason serum analysis could evaluate inflammatory response secondary to sepsis, nevertheless there is no specific diagnosis of esophageal rupture [1], [2], [3], [4], [5], [6], [7].

Contrast tomography and esophageal x-ray contrast-enhanced are the image of choice in patients with clinical suspicion of esophageal perforation with sensitivity of 90–100 %; and not only give information about the diagnosis of esophageal rupture, but extension, and involvement of adjacent structures such mediastinum (mediastinitis), pleural (pneumothorax, hydrothorax, or pleural effusions), or peritoneum; and also rule out other possible diagnosis that could mimic BS such acute aortic syndrome [7]. In our case, Chest and neck CT were performed, and the diagnosis was confirmed excluding other causes, and evaluating the extension, with no signs of extension to the mediastinum or pleural space [7]. As well, an esophageal contrast-enhanced x-ray was performed, with no evidence of a leak.

Treatment could be either non-operative (NOM) or operative including surgical, percutaneous, or endoscopically. NOM should be considered in stable patients with early presentation (<24 h), absence of surrounding spaces contamination, and contained the leak [7]. Recently, the Pittsburgh score (PS) has been developed and validated across some studies [9], [10], [11] for esophageal perforation more specifically related to spontaneous rupture, Schweigert et al. [11], demonstrate a correlation between a higher value of PS and mortality, patients with <2 points have mortality rates <3 %, compared to patients with 3–5 points with mortality of 7 %; and patients at high risk (PS score > 5), have the highest mortality probability of >37 %. This not only reflects an adequate classification but also helps guide the treatment. For patients with <2 points with a contained leak, NOM could be performed; and in cases of uncontained leak, endoscopic management could be offered. In cases of PS of 3–5, esophageal pre-existing conditions should be excluded. In patients with a contained leak, medical treatment should be offered, and clinical evaluation should be performed sequentially in order to exclude clinical signs of sepsis. In patients with uncontained leaks, operative management should be performed either with emergency esophagectomy or primary repair depending on each patient. And in high-risk patients with PS > 5 points, patient nutritional and functional status must be evaluated in order to define aggressive treatment and surgical approach. In our case, the patient meets the criteria for medical treatment according to the PS score. The surgical approach depends on the location of the perforation, due to the anatomical issues of each section of the esophagus [7], [9], [10], [11].

Conservative treatment includes nil per os, broad-spectrum antibiotics that cover anaerobic and aerobic bacteria, and proton pump inhibitor must be indicated [7]. As well nutritional support must be initiated either enteral or parenteral way to accelerate esophageal healing [7]. In cases of infected collections, percutaneous management should be preferred over surgery in stable patients [7]. In our case, medical treatment was followed, with favorable outcomes in the absence of an esophageal leak. Enteral feeding was reached after 7 days of parenteral nutrition, with any signs of sepsis.

Our case increases the existing literature regarding BS and shows the effectiveness of non-operative management in selected patients with the use of the Pittsburgh score. As well, to the best of our knowledge, this is the first case reported in Colombia.

## 4 Conclusion

Boerhaave syndrome remains to be a life-threatening condition, with high rates of morbidity and mortality. Early recognition of the disease, and effective classification of the patient in low, medium, or high risk groups according to Pittsburgh score leads to timely and targeted treatment. Multidisciplinary and strict clinical surveillance must be secured in order to achieve favorable outcomes. Psychiatric conditions must be treated in order to avoid recurrence of the disease.

## Provenance and peer review

Not commissioned, externally peer-reviewed.

## Consent

Written informed consent was obtained from the patient for publication of this case report and accompanying images. A copy of the written consent is available for review by the Editor-in-Chief of this journal by request.

## Ethical approval

Ethical committee approval (Pontificia Universidad Javeriana) was obtained in April 2023. With reference number CIE – 0021-23 and informed consent of the patient was obtained.

## Funding

This research did not receive any specific grant from funding agencies in the public, commercial, or not-for-profit sectors.

## Guarantor

Carlos Eduardo Rey Chaves.

## Research registration number

None.

## CRediT authorship contribution statement

**CERC, MD, MSc:** Participate in drafting the article and revising it critically for important intellectual content.
**MADT, MD:** Make substantial contributions to conception and design, acquisition of data, analysis and interpretation of data.
**JFIM, MD:** Make substantial contributions to conception and design, acquisition of data, analysis and interpretation of data.
**FARC, MD:** Participate in drafting the article and revising it critically for important intellectual content.
**JDR, MD, MSc:** Give final approval of the version to be submitted and any revised version.
**MCAU, MD, MSc:** Give final approval of the version to be submitted and any revised version.

## Declaration of competing interest

Authors do not declare any conflict of interest.
